# Supplementary material for: Stacking Model‐Based Classifiers for Dealing With Multiple Sets of Noisy Labels
Source: Biom J. 2025 Mar 12;67(2):e70042. doi: 10.1002/bimj.70042 (PMC11898607; doi:10.1002/bimj.70042)
Supplement: Supplementary file 1 — Supporting Information [file BIMJ-67-e70042-s001.zip › density-based-ensemble-model-main/competitors_real_data/netlab-master/netlab-master/help/somtrain.htm]

Netlab Reference Manual somtrain


# somtrain

## Purpose

Kohonen training algorithm for SOM.

## Synopsis

```
net = somtrain{net, options, x)
```

## Description

`net = somtrain{net, options, x)` uses Kohonen's algorithm to
train a SOM. Both on-line and batch algorithms are implemented.
The learning rate (for on-line) and neighbourhood size decay linearly.
There is no error function minimised during training (so there is
no termination criterion other than the number of epochs), but the
sum-of-squares is computed and returned in `options(8)`.

The optional parameters have the following interpretations.

`options(1)` is set to 1 to display error values; also logs learning
rate `alpha` and neighbourhood size `nsize`.
Otherwise nothing is displayed.

`options(5)` determines whether the patterns are sampled randomly
with replacement. If it is 0 (the default), then patterns are sampled
in order. This is only relevant to the on-line algorithm.

`options(6)` determines if the on-line or batch algorithm is
used. If it is 1
then the batch algorithm is used. If it is 0
(the default) then the on-line algorithm is used.

`options(14)` is the maximum number of iterations (passes through
the complete pattern set); default 100.

`options(15)` is the final neighbourhood size; default value is the
same as the initial neighbourhood size.

`options(16)` is the final learning rate; default value is the same
as the initial learning rate.

`options(17)` is the initial neighbourhood size; default 0.5\*maximum
map size.

`options(18)` is the initial learning rate; default 0.9. This parameter
must be positive.

## Examples

The following example performs on-line training on a SOM in two stages:
ordering and convergence.

```
net = som(nin, [8, 7]);
options = foptions;

% Ordering phase
options(1) = 1;
options(14) = 50;
options(18) = 0.9;  % Initial learning rate
options(16) = 0.05; % Final learning rate
options(17) = 8;    % Initial neighbourhood size
options(15) = 1;    % Final neighbourhood size
net2 = somtrain(net, options, x);

% Convergence phase
options(14) = 400;
options(18) = 0.05;
options(16) = 0.01;
options(17) = 0;
options(15) = 0;
net3 = somtrain(net2, options, x);
```

## See Also

`kmeans`, `som`, `somfwd`

---

**Pages:**
Index


---

Copyright (c) Ian T Nabney (1996-9)
